# Illinois State Medical Society

**Published:** 1870-08

**Authors:** N. S. Davis

**Affiliations:** Permanent-Secretary pro tem.


### ILLINOIS STATE MEDICAL SOCIETY. TWENTIETH ANNIVERSARY MEETING, HELD IN DIXON, MAY 17TH AND 18TH, 1870.

The delegates and members of the Society assembled in the Court House, and were called to order at10½ A. M., by Dr. G. W. Albin, of Neoga, 1st Vice-President, who, in the absence of the President, took the chair.

Dr. O. Evarts, Chairman of the Committee of Arrangements, welcomed the members of the Society to the hospitalities of the profession and the people of Dixon; and Dr. J. C. Corbus, Secretary of the same Committee, read the following list of the names of those who were present and registered by the Committee:

*Delegates.*

Drs. N. S. Davis, R. N. Isham.—*Chicago Medical College.*

Drs. James M. Morse, M. H. McClelland.—*Military Tract Medical Association.*

Drs. James Murphy, J. Perine Johnson, Jas. L. Hamilton. —*Peoria City Medical Association.*

Dr. David Prince.—*Morgan County Medical Society.*

Dr. R. G. Bogue.—*Chicago Medical Society.*

Dr. D. L. Holmes.—*Chicago Charitable Eye and Ear Infirmary.*

Dr. D. L. Crist.—*McLean County Medical Society.*

Drs. G. W. Phillips, G. W. Hewitt.—*Lee County Medical Society.*

Dr. Samuel J. Jones.—*St. Luke's Hospital of Chicago.*

Drs. E. P. Cook, J. C. Corbus.—*Union Pathological Society of La Salle and Adjoining Counties.*

*Permanent Members.*

Dr. G. W. Albin, of Neoga.

Dr. E. R. Willard, of Wilmington.

Dr. R. S. Higgins, of Vandalia.

Dr. James S. Whitmire, of Metamora.
Drs. Charles Hunt, O. Evarts, of Dixon.

The Committee of Arrangements also recommend for election as permanent members: Drs. T. W. Miller and J. F. Williams, of Chicago, and Dr. J. W. Hinsey, of Yates City, and they were unanimously elected.

A communication was received and read from Prof. J. V. Z. Blaney, President of the Society, stating that severe illness alone prevented his being present at the meeting.

Dr. N. S. Davis explained the absence of the Permanent Secretary, and was requested to act in his place by a vote of the Society.

The Treasurer being also absent, on account of sickness, Dr. E. P. Cook, of Mendota, was appointed Treasurer *pro tem.*

Drs. W. W. Wynne, Henry E. Payne, D. W. Law, and G. A. Bardwell, of Dixon, were duly proposed and elected permanent members.

Reports from the Standing Committees being in order, were called, with the following result:—

*Committee on Practical Medicine and Epidemics.*—Dr. C. Goodbrake, Chairman, absent.

*Committee on Surgery.*—Dr. Moses Gunn, Chairman, absent, but sent an explanation that he had a report partly prepared, and asked leave to finish it, and transfer it directly to the Committee of Publication. A motion was made to grant the request, but it being objected to on the part of several members that such a course would afford a bad precedent, the matter was referred to the Committee on Nominations.

*Committee on Obstetrics.*—Dr. Elias Wenger, Chairman, no report.

*Committee on Drugs and Medicines.*—Dr. Charles Hunt, Chairman; report ready and made the special order for $2\frac{1}{2}$ o'clock P.M.

*Committee on Necrology.*—Dr. J. H. Hollister, Chairman; no report, on account of severe and protracted sickness.

SPECIAL COMMITTEES.

*On Criminal Abortion.*—Dr. DeLaskie Miller, Chairman; no report.

*On Ophthalmology.*—Dr. J. S. Hildreth, Chairman; no report. Dr. E. L. Holmes, a member of the same Committee, had prepared a report, which was made the special order for 3½ o'clock P.M.

*On Pulmonary Tuberculosis.*—Dr. J. P. Ross, Chairman; no report.

*On the use of Plaster of Paris in Fractures.*—Dr. R. G. Bogue, Chairman; report ready, and made the special order for 5 o'clock P.M.

*On Otology.*—Dr. Samuel J. Jones, Chairman; report ready, and it was made the special order for 11 o'clock A.M., to-morrow.

*On Communication from the American Medical Association.*—Dr. N. S. Davis, Chairman; report ready, and made the special order for 10 o'clock A.M., to-morrow.

On motion of Dr. N. S. Davis, a Special Committee of three were appointed to revise and publish a new edition of the Constitution and By-Laws. The President appointed the two Secretaries and Treasurer as such Committee.

On motion of Dr. N. S. Davis, the annual assessment for the expenses of the Society was fixed at $3.00.

On motion of Dr. S. J. Jones, the Society adjourned to 2 o'clock P.M.

AFTERNOON SESSION.

The Society was called to order at 2 o'clock P.M., Dr. G. W. Albin in the Chair.

The members of the Society for the several Counties having selected their candidates for the Nominating Committee, the names were read by the Secretary as follows:—

COMMITTEE ON NOMINATIONS.

G. W. Albin, M.D., of Cumberland County.
R. T. Higgins, M.D., of Fayette County.
J. M. Morse, M.D., of Knox County.
J. C. Corbus, M.D., of La Salle County.

S. J. Jones, M.D., of Cook County.
John Murphy, M.D., of Peoria County.
E. R. Willard, M.D., of Will County.
James S. Whitmire, M.D., of Woodford County.
D. L. Crist, M.D., of McLean County.
D. E. Foote, M.D., of Boone County.
D. Prince, M.D., of Morgan County.
G. W. Phillips, M.D., of Lee County.
J. O. Hamilton, M.D., of Jersey County.
J. P. McLanahan, M.D., of Mercer County.
W. D. Sterling, M.D., of Warren County.
C. C. Lattimer, M.D., of Bureau County.
D. W. Young, M.D., of Kane County.
D. A. Sheffield, M.D., of Jo Davies County.
Samuel C. Plummer, M.D., of Rock Island County.
M. W. Walton, M.D., of Stephenson County.
H. C. Donaldson, M.D., of Whitesides County.

Dr. Evarts, Chairman of the Committee of Arrangements, introduced to the Society Col. Dement, Mayor of the city of Dixon, who, after some very appropriate remarks, extended an invitation to attend a *Picnic* in Mr. Charter's Grove, on the afternoon of Wednesday, and a party at the Wachusett House in the evening. The invitation was given in behalf of the city authorities and citizens of Dixon.

The Vice-President, Dr. Albin, responded cordially to the Mayor; and, on motion, the proffered hospitalities were accepted.

The Secretary of the Committee of Arrangements reported the names of several physicians as suitable for election as permanent members, and they were unanimously elected as follow:—

D. A. Sheffield, M.D., of Apple River, Ill.
R. W. Shaw, M.D., of Ashton, Ill.
E. R. Travers, M.D., of Amboy, Ill.
J. B. Felker, M.D., of Amboy, Ill.
C. C. Lattimer, M.D., of Princeton, Ill.
G. W. Crossley, M.D., of Princeton, Ill.

The Secretary of the same Committee reported the names of the following delegates and permanent members as having registered since previous report:

M. M. Robbins, M.D., of Aurora, Fox River Valley Medical Association.

Geo. J. Munroe, M. D., of Leland, Medico-Pathological Society.

Edwin Powell, M.D., of Chicago, Rush Medical College.

D. W. Young, M. D., of Aurora, Fox River Valley Medical Association.

Sam'l C. Plummer, M.D., of Rock Island, Iowa and Illinois Central Dist. Medical Society.

Jno. P. McGlanahan, M.D., of Norwood, Military Tract Medical Society.

W. D. Sterling, M.D., ———, Military Tract Med. Society.

D. E. Foote, M.D., of Belvidere, permanent member.

J. O. Hamilton, M.D., of Jerseyville, permanent member.

Dr. Chas. Hunt, of Dixon, Chairman of the Committee on Drugs and Medicines, read his report, and the same was received and referred to the Committee of Publication.

Dr. E. L. Holmes, of Chicago, a member of the Committee on Ophthalmology, read a report on the conjunctivitis of infants. The reporter recommended the use of strong solution of nitras argenti in the early stage of the disease.

Dr. D. Prince alluded to the contagious character of the disease, and inquired in reference to the success of compression as a remedy, as advocated by Dr. Hildreth.

Dr. E. L. Holmes replied that recent investigations in large hospitals, had shown that poisoning of the eyes of infants from the vaginal discharge of the mother is not of frequent occurrence. He thought that ordinary catarrhal conjunctivitis, in infants, was very prone to assume a purulent character. In regard to treatment by compression, he would only say that recent authors of the best character do not recommend it. In reply to a question by Dr. Johnson, of Peoria, Dr. Holmes said he would use the strong nit. silver solut. in cases involving ulceration of cornea and danger of sloughing.

Dr. J. P. Johnson, of Peoria, thought the nervous sensibility was less in infants than in adults. He strongly recommended the application of the liquid carbolic acid to the ulcers of the cornea, connected with infantile and purulent ophthalmia, claiming that he had derived the most prompt benefit therefrom.

Dr. S. J. Jones, of Chicago, expressed the opinion that more benefit was derived from scarification applied to the tumefied conjunctiva, than from compression.

After some remarks from Dr. Isham, of Chicago, the report of Dr. Holmes was referred to the Committee of Publication.

Dr. R. G. Bogue read a report on the use of plaster of Paris in the treatment of fractures, which was received and referred to the Committee of Publication.

Dr. Jas. S. Whitmire, of Metamora, one of the members of the Committee on Practical Medicine, read a report on the use of hydrate of chloral in the treatment of cerebro-spinal meningitis; detailing two cases that had been successfully treated in his practice. The report was received and referred to the Committee of Publication.

Dr. D. W. Young, of Aurora, stated that he had written a paper and sent it to the Chairman of the Committee on Practical Medicine, on the practice of venesection in the treatment of pneumonia; but the chairman of the committee being absent, the paper could not be presented to the Society at this time. On motion, Dr. Young was requested to furnish a copy of his paper to the Committee of Publication.

The Special Committee on Criminal Abortions, Dr. DeLaskie Miller, of Chicago, Chairman, made no report.

The Special Committee on Pulmonary Tuberculosis, Dr. J. P. Ross, of Chicago, Chairman, made no report.

Dr. D. W. Young, of Aurora, called up the subject of scarlet fever, and an interesting discussion followed, participated in by Drs. D. W. Young, S. J. Jones, D. A. Sheffield, D. L. Crist, D. Prince, N. S. Davis, E. P. Cook, J. C. Corbus, and R. N. Isham.

From the remarks of these gentleman it was evident that scarlet fever had been more prevalent and severe during the preceding year than usual. But no new facts of importance were elicited in regard to the pathology or treatment.

Dr. D. W. Young proposed the use of carbolic acid in suppurating wounds, etc., as a profitable subjeet for discussion during the evening session.

Dr. N. S. Davis spoke of the great importance of keeping written records of cases and their results, alleging that when we trust simply to our general impressions in regard to the results of management in any given form of disease, we are liable to error. He adduced some examples to illustrate his position. The Society adjourned to 8 o'clock P.M.

EVENING SESSION.

The members were called to order at 8 o'clock P.M., Dr. G. W. Albin, Vice-President, in the chair.

The Secretary of the Committee of Arrangements reported the registration of Drs. D. M. Vosburgh, of Earlville, and H. C. Donaldson, of Monmouth, as permanent members. The same Committee recommended the election as permanent members of Drs. Chas. McAllister, of Dixon, M. W. Walton, of Ridott, and Thos. Winston, of Forreston. On motion, they were unanimously elected.

The value of carbolic acid in the treatment of wounds was taken up for consideration. Dr. R. N. Isham, of Chicago, remarked that he did not apply carbolic acid to freshly incised wonnds, and does not regard it as beneficial in such cases, where the arrest of hemorrhage and the perfect apposition of wounded surfaces are the principal objects. In contused wounds and compound fractures, in which union is not expected, the acid is valuable as an anticeptic. But in these latter cases, especially when involving gangrene and extensive suppuration, he prefers iodoform, a compound of iodine and chloroform.

Dr. R. G. Bogue, of Chicago, stated that after the introduction of carbolic acid into surgical practice, it was used extensively in the Cook County Hospital, for one year or more, but with such results that its subsequent use has been much more limited. In many of the cases in which it was applied to flaps and stumps after amputation, it appeared to dry up the surfaces and prevent healthy granulation. Injected into large abcesses, or applied to copiously suppurative surfaces, it lessened the formation of pus, and appeared to do good. At present he did not apply it to the surface of incised or contused wounds, but only as a covering or external dressing.

Dr. D. W. Young, of Aurora, said his experience had been similar to that of Dr. Bogue; but in addition said he had seen a few cases in which he thought it had induced blood-poisoning, and in one case, after it had been used eight or ten days, extensive phlebitis followed, causing death of the patient.

Dr. D. Prince, of Jacksonville, thought the efficacy of carbolic acid and other antiseptics depended on their power to destroy parasitic life. And the practical question to determine is, what strength of these agents will prevent or destroy parasitic life, without injuring the surfaces to which they are applied, or proving detrimental to the patient by absorption. Carbolic acid used of the strength of only three or four grains to the ounce of water, does not prevent union or granulations, but does prevent suppuration and parasitic germs. When applied to old suppurating surfaces, he thought it might be used with benefit much stronger. But when applied to wounds of cut surfaces strong enough to dry the parts or make them look pale or blue, it was injurious and unnecessarily strong. He recommended for application outside of isinglass plasters, a mixture of equal parts of Calvert's solution of carbolic acid No. 4, with a solution of chloride of zinc and glycerine. He thought it not only capable of preventing the dressings from becoming offensive, but also lessening the tendency to suppuration in the wound. He thought a weak solution might be used with advantage as a vaginal wash in obstetrical practice. He also recommended a solution of 16 gr. to the ounce of water, to be used in the form of spray with the atomizer, in cases of putrid sore throat.

Dr. Prince also stated that he had once injected undiluted Calvert's solution of carbolic acid No. 4 into a nevus, and at another time into a lymphatic gland. In both instances it produced extreme paleness of countenance, feebleness of pulse, coldness of extremities, etc., from which he inferred it to act as a direct sedative upon the system.

Dr. John Murphy, of Peoria, said he had used the acid in almost every stage of suppurative action, with but little benefit; but that its application to recent wounds had been very valuable in preventing suppuration. He used it of the strength of one drachm and one drachm and a-half to the pint of water.

Dr. D. E. Foote, of Belvidere, inquired whether the acid might be used with effect for the destruction of parasites in the stomach and intestines.

Dr. J. S. Whitmire, of Metamora, thought when the acid had acted injuriously on wounds, or with poisonous effect on the system, it was from using it too strong.

Dr. N. S. Davis, of Chicago, stated that he had very little experience in the use of the acid as an application to wounds, but had used it in a variety of cases as an internal remedy. He had found it of great value in arresting vomiting in some cases of pregnancy, and in many cases of gastric irritability, both in children and adults. He had given it with some *apparent* advantage in three or four cases of cancerous disease of the stomach and uterus.

Dr. D. L. Crist, of Bloomington, had used it with advantage in cases of gastric irritation and vomiting.

Dr. J. P. Johnson, of Peoria, had used it in the treatment of wounds, with advantage; also as a local application in syphilitic ozena; and in purulent discharges from the ear.

On motion, the Society adjourned to 9 o'clock A.M.

SECOND DAY.—MORNING SESSION.

The Society was called to order at 9½ o'clock A.M., Dr. G. W. Albin, Vice-President, in the chair.

The Committee of Arrangements presented the following additional names of delegates:

Prof. E. Andrews, of Chicago, from Mercy Hospital.

Prof. H. A. Johnson, of Chicago, from Mercy Hospital.

Prof. E. Ingalls, of Chicago. from Rush Med. College.

The same Committee also reported the following, who were unanimously elected permanent members:

T. L. Carey, M.D., of Lena, Ill.
M. B. Campbell, M.D., of Wilmington, Ill.
W. M. Kaul, M.D., of Limerick, Bureau county, Ill.
J. R. Zearing, M.D., of Dover, Bureau county, Ill.

Dr. N. S. Davis, in behalf of Dr. J. H. Hollister, Treasurer, presented the following report of the receipts and expenditures for the yast year:

*Annual Report of the Treasurer of Illinois State Medical Society, for the year ending May,* 1870.

Your Treasurer herewith submits his *Ninth* Annual Report, as follows:

J. H. HOLLISTER, *Treasurer*,
In acc't with ILL. STATE MED. SOCIETY.

*Dr.*

| | |
|---|---|
| To Balance in the Treasury May, 1869, | $ 86 00 |
| Dues paid by 60 members at the Annual Meeting in Chicago, | 240 00 |
| Dues by mail as result of personal correspondence, | 80 00 |
| Total | $406 00 |

*Cr.*

| | |
|---|---|
| By account paid Fergus' Sons for printing Transactions for 1869, | 199 06 |
| Balance in the Treasury May, 1870, | $206 94 |

Dr. S. J. Jones, of Chicago, read his report on Otology, exhibiting therewith an interesting series of instruments used for the diagnosis and treatment of diseases of the ear.

On motion the report was received and referred to the Committee of Publication.

Dr. E. L. Holmes, one of the Committee on Ophthalmology, also read a paper on conjunctival inflammation in infants.

Dr. J. P. Johnson, of Peoria, confirmed the statement in the paper, that the use of the nasal douche, used with too much pressure endangered inflammation in the ear; and mentioned a case in which the tympanum was ruptured, and the water discharged through the ear.

Dr. H. A. Johnson, of Chicago, remarked in relation to the nasal douche, that there were two dangers requiring the attention of the practitioner.

First, too high a pressure; and Second, forcible efforts to expel the mucus from one nostril while the wash is passing. He thought the latter more frequently the cause of trouble than the former; but regarded the nasal douche, when properly used, of great value.

Dr. S. J. Jones, agreed with the last speaker, but in many instances preferred the curved nasal syringe instead of the douche.

Dr. E. L. Holmes said patients sometimes have a spasmodic action during the use of the nasal douche, causing great pressure on the ears, which it is difficult to guard against.

Dr. J. P. Johnson, of Peoria, regarded the use of a nasal atomizer as preferable, in some cases, to the douche.

On motion, the paper of Dr. Holmes was referred to the Committee of Publication.

Dr. N. S. Davis, Chairman of the Committee, to whom was referred a communication from the American Medical Association, made a report accompanied by resolutions. [See report in full in another part of the Transactions.]

Dr. E. Ingalls, of Chicago, offered the following resolution:

*Resolved*, That we acknowledge with gratitude and pride the advance the medical profession has made and is continually making in knowledge and scientific attainments; and that practical skill, which enables its practitioners successfully to combat disease and soothe the suffering of the sick; and that in the labor which has effected this advance the Medical Colleges of the country have borne an honorable part, and have contributed their full influence; and that the Illinois State Medical Society would urge the trustees and faculties of all such colleges to carry forward this work of improvement in the spirit they have formerly evinced, and to be always dilligent in the future as in the past, to make such changes in the course and scope of teaching, as the light of experience may from time to time suggest to their good judgments; and so far as we are able we will discard all partisan and personal feelings, and give our approbation and support to such Colleges as impart the most thorough, extensive, and correct teaching.

The subject was discussed by Drs. E. Ingalls, D. W. Young, J. S. Whitmire, and N. S. Davis; when, on motion, the report and resolutions were acepted, and referred to the Committee of Publication. The resolution offered by Dr. Ingalls was also referred to the Committee of Publication.

Dr. O. Evarts, Chairman of the Committee of Arrangements, announced the time for the *Picnic* in Gov. Charter's Grove, from 1½ to 6 o'clock P.M.

The Committee on Nominations reported the following names of officers and committees for the ensuing year:

*President*—G. W. Albin, M.D., of Neoga.

*1st Vice-President*—John Murphy, M.D., of Peoria.

*2d Vice-President*—J. S. Whitmire, M.D., of Matamora.

*Treasurer*—J. H. Hollister, M.D., of Chicago.

*Permanent-Secretary*—T. D. Fitch, M.D., of Chicago.

*Assistant-Secretary*—J. P. Johnson, M.D., of Peoria.

*Committee of Arrangements*—Drs. R. Boal; J. L. Hamilton; R. Roskoton, of Peoria; S. Moss, of Pekin; and J. S. Whitmire, of Matamora.

Standing Committees:—

*On Practical Medicine*—Drs. H. A. Johnson, of Chicago; J. C. Corbus, of Mendota; and N. Wright, of Chatham.

*Committee on Surgery*—Drs. E. Andrews, of Chicago; 'D. Prince, of Jacksonville; Webster, of Monmouth.

*On Obstetrics*—Drs. DeLaskie Miller, of Chicago; John P. McClanahan, of Norwood; and O. Everts, of Dixon.

*On Drugs and Medicines*—Drs. E. Ingalls, of Chicago; B. Griffith, of Springfield; and M. Shepherd, of Payson.

Special Committees:—

*On Criminal Abortions*—Drs. W. H. Byford, of Chicago; Chas. Hunt, of Dixon; and D. E. Foote, of Belvidere.

*On Ophthalmology*—Drs. H. H. Roman, of Springfield; J. P. Johnson, of Peoria; and E. L. Holmes, of Chicago.

*On Phthisis Pulmonalis*—Drs. S. W. Noble, of Bloomington; D. H. Law, of Dixon; and J. Adams Allen, of Chicago.

*On Otology*—Drs. S. J. Jones, of Chicago; Thos. Winston, of Forreston; and J. T. Frazier, of Howard's Point.

*On Criminal Insanity*—Drs. A. McFarland, of Jacksonville; J. S. Whitmire, of Matamora; and R. J. Patterson, of Batavia.

*On Idiocy*—Drs. C. T. Wilbur, of Jacksonville; J. R. Gilmore, of Jacksonville; and H. Noble, of Heyworth.

*On Medical Uses of Carbolic Acid*—Drs. N. S. Davis, of Chicago; D. H. Law, of Dixon; and John Murphy, of Peoria.

*On Cholera Infantum*—Dr. D. W. Young, of Aurora.

*On Membranous Croup*—Dr. D. Prince, of Jacksonville.

*On Gleet*—Dr. R. L. Higgins, of Vandalia.

*On Trichiasis*—Dr. J. S. Hildreth, of Chicago.

*On Results in Fractures*—Dr. G. W. Phillips, of Dixon.

*On Intestinal Affections of Infants*—Dr. T. D. Fitch, of Chicago.

*On Diabetes*—Dr. W. D. Sterling, of Ionia.

*On Perineal Section*—Dr. E. Powell, of Chicago.

*On Duties of the Profession to the State Medical Society*—Dr. J. S. Whitmire, of Matamora.

*On Causes of Mortality after Amputations*—Dr. R. N. Isham, of Chicago.

For next place of Annual Meeting of the Society, Peoria, on the third Tuesday in May, 1871.

On motion, the report of the Committee was accepted and adopted.

The Society then adjourned until 7 o'clock P.M.

Note. During the afternoon, the members of the Society were entertained very pleasantly in the Grove of Gov. Charter's on the bank of Rock River, by the profession and people of Dixon. The Mayor of the city, Col. Demant, received the Society on behalf of the government and citizens of Dixon, and was followed in response by Drs. N. S. Davis, H. A. Johnson, E. Ingalls, O. Evarts, and Rev. Williams.

The ladies contributed largely to the pleasure of the occasion, both by their presence and their baskets of abundant refreshments.

EVENING SESSION.

The Society was called to order at 8 P.M. Dr. G. W. Albin in the chair.

Dr. E. Ingalls gave a verbal synopsis of a paper hehad partially prepared, on the causes, preventions and treatment of Scarlatina.

On motion, he was requested to complete the paper and present it to the Committee of Publication.

Dr. J. C. Corbus presented a report, as one of the Committee on Otology, which was referred to the Committee of Publication.

Dr. E. Powell, of Chicago, reported a case of Amputation at the Hop-Joint, which was referred to the Committee of Publication.

The Committee on Nominations recommended that Dr. Moses Gunn, Chairman of the Committee on Surgery for the past year, and Dr. DeLaskie Miller, Chairman of the Committee on Criminal Abortion for the same time, be requested to complete their reports and transfer them to the Committee of Publication. On motion the recommendation of the Committee was adopted.

On motion, the Committee on Necrology, Dr. J. H. Hollister, of Chicago, Chairman, was continued.

The following resolution was offered by Dr. J. S. Whitmire, and adopted.

*Resolved*, That it is the duty of the Legislature of the State of Illinois, to make such legal provisions as to insure persons who have committed capital crimes, and been cleared of the same on account of the plea of Insanity, being sent to the Lunatic Asylum, as being dangerous members of Society; and that a committee be appointed by this Society to procure such action.

The Society then proceeded to the election of Delegates to the American Medical Association and several State Medical Societies, as follows:

Delegates to the American Medical Association—Drs. N. S. Davis, of Chicago; G. W. Albin, of Neoga; D. W. Young, of Aurora; J. P. Johnson, of Peoria; D. L. Crist, of Bloomington; J. S. Whitmire, of Matamora; W. W. Wynne, of Dixon; M. W. Walton, of Ridot; E. P. Cook, of Mendota; J. O. Hamilton, of

Jerseyville; R. N. Isham, of Chicago; E. Ingalls, of Chicago; G. C. Paoli, of Chicago; R. J. Higgins of Vandalia; S. J. Jones, of Chicago; E. Powell, of Chicago; J. M. Morse, Galesburg; H. A. Johnson, of Chicago; G. J. Munroe, of Leland; Moses Gunn, of Chicago; M. M. Robbins, of Aurora; J. P. McLanahan, of Mecer Co.; G. W. Heath, of Franklin Grove.

Delegates to State Medical Societies:—

Iowa—Drs. S. C. Plummer, of Rock Island; and W. D. Sterling, of Ionia.

Missouri—Drs. E. P. Cook, of Mendota; and S. P. Breed, of Princeton.

Indiana—Drs. W. H. Byford and J. P. Ross, of Chicago.

Wisconsin—Drs. W. C. Lyman, of Chicago; and D. E. Foote, of Belvidere.

Kansas—Drs. G. W. Philips, of Dixon; J. M. Morse, of Galesburg; and J. S. Whitmire, of Metamora.

Ohio—Dr. Chas. Hunt, of Dixon.

On motion of Dr. H. A. Johnson, delegates who were unable to attend the meeting to which they had been appointed, were authorized to send a substitute, provided such substitute must be a member of the State Society.

Dr. D. W. Young, of Aurora, offered the following resolutions, which were unaniously adopted, and copies of the same furnished to the press of Dixon.

*Resolved*, That the thanks of this State Medical Society are hereby tendered the Mayor and Common Council, and citizens of the city of Dixon, for their hospitality and the magnificent entertainment of its Delegates, while in attendance upon the twentieth session of the Society.

*Resolved*, That the thanks of the Society are hereby tendered to the President for the able manner in which he has discharged the duties of his office; and to the Committee of Arrangements for the excellent accommodations provided for the present meeting.

*Resolved*, That the thanks of the Society are hereby tendered to the very hospitable Host of the Wachusa House; to the Illinois Central; the Chicago, Burlington and Quincy; the

Chicago, Alton and St. Louis; the Chicago and Rock Island; and the Chicago and North-Western Railroad Companies, for their liberality in commuting fares, and their excellent accommodations to members of this Society.

Dr. E. P. Cook, of Mendota, offered the following, which was adopted, and a Committee appointed consisting of Drs. J. H. Rauch and H. A. Johnson, of Chicago, and D. Prince, of Jacksonville.

*Resolved*, That it is the duty of the Legislature of the State of Illinois to make such legal enactments, or amend such laws as exist, so as to secure a perfect registration of births, marriages, and deaths, etc. in the State. And that a Committee be appointed by this Society to memoralize the Legislature at its next session, and also for legalizing dissections.

Dr. J. S. Whitmire, of Matamora, offered the following which was adopted:

*Resolved*, That it shall be the rule of this Society, that hereafter no report or paper designed for the Illinois State Medical Society, shall be referred to the Committee on Publication without first having been read in full, or a synopsis of the same, before the Society.

Dr. E. Ingalls, of Chicago, offered the following Amendments to the Constitution of the Society, which lie over to the next meeting:

Permanent Members—Amend by erasing the words "But without the right of voting."

Also. After the word "specifying" insert the words "From what source he has received his authority to practice."

Meetings-Substitute—"The time and place for each annual meeting shall be determined by vote of the Society, of which due notice shall be given by the Secretary.

Officers—From the clause defining the duties of the President, erase the words "And deliver an address at the expiration of his term of office."

Treasurer—Amend to read "He shall be a member of the Committee on Publication, through which Committee he shall present his accounts, duly authenticated, at every regular meeting.

Standing Committees—Amend by adding "a Committee on Therapeutics, a Committee on Ophthalmology and Otology, and a Committee on Hygiene."

Erase duties specified for Committees on Practical Medicine, Surgery, Obstetrics, and Drugs and Medicines, and substitute the following: "Each of these Committees shall prepare an annual report, to be submitted to the consideration and action of the Society, which report may embrace whatever the said Committees deem most important to communicate in their several departments, and especially all improvements and discoveries relating to them."

Committee on Publication—Amend to read after the words "As may be ordered to be published." "The members of the Committee shall annually audit and authenticate the accounts of the Treasurer, and present a statement of the same in their annual report, which report shall also specify the character and cost of the publications, etc."

After some appropriate remarks by the President the Society adjourned *sine die.*

N. S. DAVIS, *Permanent-Secretary pro tem.*

